# Association between temporal trajectories of inflammatory markers and prognosis in ischemic stroke

**DOI:** 10.3389/fneur.2026.1765722

**Published:** 2026-07-29

**Authors:** Jing Xu, Xiaolan Wu, Xiaorong Lu, Shijun Li

**Affiliations:** 1 Department of Neurology, Affiliated Dongyang Hospital of Wenzhou Medical University, Dongyang, China; 2 Department of Patient Services Management, Affiliated Dongyang Hospital of Wenzhou Medical University, Dongyang, China

**Keywords:** acute ischemic stroke, functional outcomes, inflammatory markers, latent class analysis, temporal trajectory

## Abstract

**Background:**

The post-stroke inflammatory response is a dynamic and heterogeneous process that cannot be fully captured by single time-point measurements. This study aimed to characterize the temporal trajectories of four inflammatory markers—neutrophil-to-lymphocyte ratio (NLR), monocyte-to-lymphocyte ratio (MLR), systemic immune-inflammation index (SII), and systemic inflammation response index (SIRI)—during the acute phase of ischemic stroke and to evaluate their associations with 3-month functional outcomes.

**Methods:**

We retrospectively analyzed patients with acute ischemic stroke admitted within 24 h of onset at Dongyang People’s Hospital from January to December 2024. Complete blood counts collected within the first 7 days were used to construct latent class growth models. Model selection was based on Akaike Information Criterion, the Bayesian Information Criterion, entropy, average posterior probability, and minimum class size. Logistic regression was performed to examine associations between inflammatory trajectories and 3-month modified Rankin Scale outcomes.

**Results:**

A total of 735 patients were included. Distinct inflammatory trajectories were identified for each marker: three classes for NLR (stable-low, rapid-increase, gradual-decrease), three for MLR (persistent-low, persistent-moderate, persistent-high), three for SII (stable-low, persistent-moderate, marked-decline with rapid-rebound), and four for SIRI (stable-low, gradual-increase, gradual-decrease, rapid-increase). Stable-low patterns constituted the majority across all markers. For NLR, the rapid-increase trajectory consistently predicted poor 3-month outcomes (Model 3: OR 3.492, 95% CI 1.514–10.265). For SIRI, both gradual-increase and rapid-increase trajectories were independently associated with poor prognosis (Model 3: OR 3.894, 95% CI 1.395–10.872; OR 4.264, 95% CI 1.201–15.136). In contrast, MLR and SII trajectories were not associated with functional outcomes after full adjustment.

**Conclusion:**

Distinct temporal inflammatory trajectories are observed during the 1 week after ischemic stroke. Increasing trajectories of NLR and SIRI strongly predict poor 3-month outcomes. These findings underscore the clinical importance of monitoring early inflammatory evolution rather than relying on single measurements and provide a novel framework for prognostic assessment in ischemic stroke.

## 1 Introduction

Ischemic stroke (IS) remains a major global public health challenge (1). It is the second leading cause of death worldwide and the third leading cause of death and disability, as measured by disability-adjusted life years (2). Cerebral ischemia and hypoxia lead to neuronal necrosis and the release of damage-associated molecular patterns (DAMPs) (3, 4). These molecules bind to receptors on immune cells, stimulating microglia and peripheral immune cells to secrete proinflammatory cytokines and activating the immunoinflammatory response (5). Meanwhile, disruption of the blood–brain barrier after IS increases its permeability, enabling peripheral immune cells to infiltrate brain tissue and further amplifying intracranial inflammation (6).

During the acute phase of cerebral infarction (approximately the first 7 days), the inflammatory response typically progresses through three stages (4, 7). In the initiation phase (0–6 h), ischemia-induced neuronal necrosis releases DAMPs, triggering early microglial activation, mild endothelial adhesion molecule expression, initial neutrophil infiltration, and low-level pro-inflammatory cytokine expression (4, 8). The rapid recruitment phase (6–24 h) is characterized by massive neutrophil and monocyte infiltration, elevated chemokines, enhanced microglial and astrocyte pro-inflammatory activity, and early peripheral immune suppression (3, 7). In the peak and plateau phase (2–7 days), inflammatory markers reach their highest levels, neutrophils and monocytes infiltrate the ischemic core, adaptive immune cells including T and B cells accumulate, regulatory T cells upregulate anti-inflammatory cytokines, and neutrophil-derived reactive oxygen species and matrix metalloproteinase-9 contribute to blood–brain barrier disruption and secondary brain injury (6, 9). Excessive activation of the immunoinflammatory response aggravates cytotoxic edema and accelerates the conversion of penumbral tissue into the core infarct zone (5, 10). In severe infarction, cerebral ischemia–induced immunosuppression syndrome produces a pathological imbalance characterized by intracranial hyperinflammation and systemic immunosuppression, increasing the risk of complications such as pneumonia and urinary tract infection (11). After the acute phase, autoreactive T cells may further target normal or reparative neural tissue, impair synaptic remodeling, and elevate the risk of post-stroke dementia (12).

A substantial body of research has examined the association between inflammatory markers and outcomes after cerebral infarction. Evidence indicates that both conventional inflammatory markers—such as C-reactive protein and inflammatory prognostic index—and novel markers, including cytokines and matrix metalloproteinase-9, correlate with stroke prognosis (7, 13, 14). However, most studies rely on single time-point measurements, and the prognostic value of dynamic changes in inflammatory markers during the acute phase of IS remains unclear. Considering the post-stroke inflammatory response is a complex and evolving process, single time-point measurements cannot adequately reflect the inflammatory status during the acute phase. Therefore, in this study, we aimed to investigate the temporal trajectories of inflammatory markers, including neutrophil-to-lymphocyte ratio (NLR), monocyte-to-lymphocyte ratio (MLR), systemic immune-inflammation index (SII), and systemic inflammation response index (SIRI), during the acute phase of cerebral infarction and to evaluate their associations with 3-month modified Rankin Scale (mRS) scores.

## 2 Materials and methods

### 2.1 Patients

In this study, we retrospectively analyzed consecutive patients with acute ischemic stroke (AIS) admitted to Dongyang People’s Hospital between January 1, 2024, and December 31, 2024. The study was approved by the Ethics Committee of Dongyang People’s Hospital and conducted in accordance with the principles of the Declaration of Helsinki. Patients were included if they met the following criteria: (1) age ≥18 years; (2) diagnosis of AIS according to the Chinese AIS Diagnosis and Treatment Guidelines (15); (3) admission within 24 h of symptom onset; and (4) written informed consent provided by the patient or their legally authorized representative. The exclusion criteria were: (1) patients who underwent endovascular intervention; (2) patients with autoimmune or hematological disorders; (3) presence of acute or chronic infections; (4) use of corticosteroids or immunosuppressive agents during hospitalization; (5) premorbid modified Rankin Scale (mRS) score >2; (6) patients with only a single inflammatory marker measurement within the first 7 days after admission; and (7) incomplete or missing clinical data, including unavailable 3-month functional outcome assessments.

### 2.2 Definition of inflammatory markers

The NLR is calculated as the number of neutrophils divided by the number of lymphocytes; the MLR is calculated as the number of monocytes divided by the number of lymphocytes; the SII is calculated as platelets multiplied by neutrophils and divided by lymphocytes; and the SIRI is calculated as monocytes multiplied by neutrophils and divided by lymphocytes (16).

### 2.3 Data collection

Upon admission, nurses collected patients’ demographic data, including age, sex, height, and weight, and measured blood pressure using an electronic sphygmomanometer. Neurologists assessed neurological function at admission using the National Institutes of Health Stroke Scale (NIHSS) and mRS. At discharge, stroke etiology was determined according to the Trial of Org 10172 in Acute Stroke Treatment (TOAST) classification (17), based on examinations conducted during hospitalization. Hypertension, diabetes mellitus, ischemic heart disease, and atrial fibrillation were considered present if documented in medical records or confirmed at discharge. A history of stroke was defined as a prior transient ischemic attack or stroke. Complete blood counts (CBC) were performed using an automated hematology analyzer (XN series). CBC were routinely collected at admission and discharge. Depending on the patient’s condition, serial blood tests were performed during hospitalization, and all available CBC results within the first 7 days of admission were included for analysis. Patients were followed up 3 months (±1 week) after stroke onset via outpatient visits or telephone. Functional outcomes were evaluated using the mRS, with scores ≥3 indicating poor prognosis and scores <3 indicating favorable prognosis.

### 2.4 Statistical analysis

Latent class growth models (LCGM) were constructed using the lcmm package in R to identify distinct trajectories of inflammatory markers. As repeated CBC measurements were mostly 2–4 per patient, we first specified a single-class quadratic growth model with days as the time scale, then incrementally increased the number of classes to construct models containing 2–5 classes. Model selection was based on the Akaike Information Criterion (AIC), Bayesian Information Criterion (BIC), entropy, and average posterior probability (APP). The optimal number of classes was determined based on entropy and APP greater than 0.8, lower information criterion values, and a minimum class size of at least 1% of the total population to ensure adequate statistical power, and clinically meaningful trajectory patterns. Continuous variables are presented as medians with interquartile ranges (IQRs) after testing for normality using the one-sample Shapiro–Wilk test. Categorical variables are expressed as counts and percentages. The Kruskal–Wallis test was used for continuous variables across classes, and the Pearson chi-square test or Fisher’s exact test was applied for categorical variables, as appropriate. Three logistic regression models were further constructed to examine potential associations between inflammatory trajectories and stroke outcomes. Three logistic regression models were constructed with sequential adjustment for potential confounders. Model 1 was unadjusted; Model 2 adjusted for age, sex, hypertension, and diabetes mellitus; Model 3 additionally adjusted for NIHSS score, systolic blood pressure, intravenous thrombolysis, Low-Density Lipoprotein, HbA1c, and TOAST classification. All analyses were performed using R version 4.5.1 and SPSS version 26. Two-sided *p* values <0.05 were considered statistically significant.

## 3 Results

### 3.1 Model fit and selection for LCGM

The goodness-of-fit statistics for the LCGM models are presented in Tables 1, 2. For both the NLR and MLR models, AIC and BIC decreased progressively as the number of classes increased. However, in the NLR models, the entropy for the 4-class solution fell below 0.80, the APP of one class in the 5-class model was 0.665, indicating poor class separation. In the MLR models, the entropy for both the 4-class and 5-class solutions was below 0.80, and therefore the 3-class model was considered the optimal solution. For SII, the entropy reached 1.0 in the 5-class model, suggesting overfitting. Among the remaining models, the 3-class model exhibited the lowest AIC and BIC values and was therefore selected. For SIRI, the 4-class model had the lowest AIC and BIC values among all candidate models, and all other fit indices met the predefined criteria, indicating that the 4-class model was the most appropriate.

**Table 1:** Fit statistics for four inflammatory marker trajectories.

Inflammation markers	Number of classes	AIC	BIC	Entropy	%class 1	%class 2	%class 3	%class 4	%class 5
NLR	1	7684.4	7702.8	1.000	100.00				
	2	7244.7	7281.5	0.873	85.31	14.69			
	3	7142.9	7198.1	0.903	82.99	4.63	12.38		
	4	7114.9	7188.5	0.784	74.56	12.38	2.86	10.20	
	5	7061.5	7153.5	0.843	9.52	77.82	5.71	4.49	2.45
MLR	1	−1643.4	−1625.0	1.000	100.00				
	2	−2278.9	−2242.1	0.955	92.38	7.62			
	3	−2497.9	−2442.7	0.839	21.09	75.51	3.40		
	4	−2539.1	−2465.5	0.785	6.12	20.14	70.07	3.67	
	5	−2620.2	−2528.2	0.797	1.77	4.76	68.71	23.67	1.09
SII	1	29,701.0	29,719.4	1.000	100.00				
	2	29,206.6	29,243.4	0.892	88.16	11.84			
	3	29,008.0	29,063.2	0.874	79.86	18.50	1.63		
	4	29,016.0	29,089.5	0.900	79.86	0.00	18.50	1.63	
	5	20,000.0	20,000.0	1.000	0.00	0.00	0.00	0.00	0.00
SIRI	1	6518.0	6536.4	1.000	100.00				
	2	5993.4	6030.2	0.962	93.47	6.53			
	3	5811.5	5866.7	0.953	5.44	91.84	2.72		
	4	5692.0	5765.6	0.907	86.26	4.63	6.67	2.45	
	5	5724.3	5816.3	0.616	2.31	81.09	0.00	14.56	2.040

AIC, Akaike Information Criterion; BIC, Bayesian Information Criterion; NLR, Neutrophil to Lymphocyte Ratio; MLR, Monocyte to Lymphocyte Ratio; SII, Systemic Immune—Inflammation Index; SIRI, Systemic Inflammatory Response Index.

**Table 2:** Average posterior probabilities for four inflammatory marker trajectories.

Inflammation markers	Number of classes	APP 1	APP 2	APP 3	APP 4	APP 5
NLR	1	1.000				
	2	0.974	0.918			
	3	0.971	0.878	0.913		
	4	0.917	0.665	0.900	0.882	
	5	0.844	0.937	0.665	0.805	0.840
MLR	1	1.000				
	2	0.992	0.934			
	3	0.854	0.945	0.976		
	4	0.787	0.756	0.916	0.948	
	5	0.766	0.935	0.896	0.831	0.960
SII	1	1.000				
	2	0.980	0.898			
	3	0.960	0.870	0.913		
	4	0.960	NA	0.870	0.913	
	5	NA	NA	NA	NA	NA
SIRI	1	1.000				
	2	0.993	0.926			
	3	0.890	0.985	0.944		
	4	0.964	0.844	0.852	0.937	
	5	0.909	0.728	NA	0.776	0.943

APP, Average Posterior Probabilities; NLR, Neutrophil to Lymphocyte Ratio; MLR, Monocyte to Lymphocyte Ratio; SII, Systemic Immune—Inflammation Index; SIRI, Systemic Inflammatory Response Index.

### 3.2 Trajectory patterns of inflammatory markers

**Figure 1:**
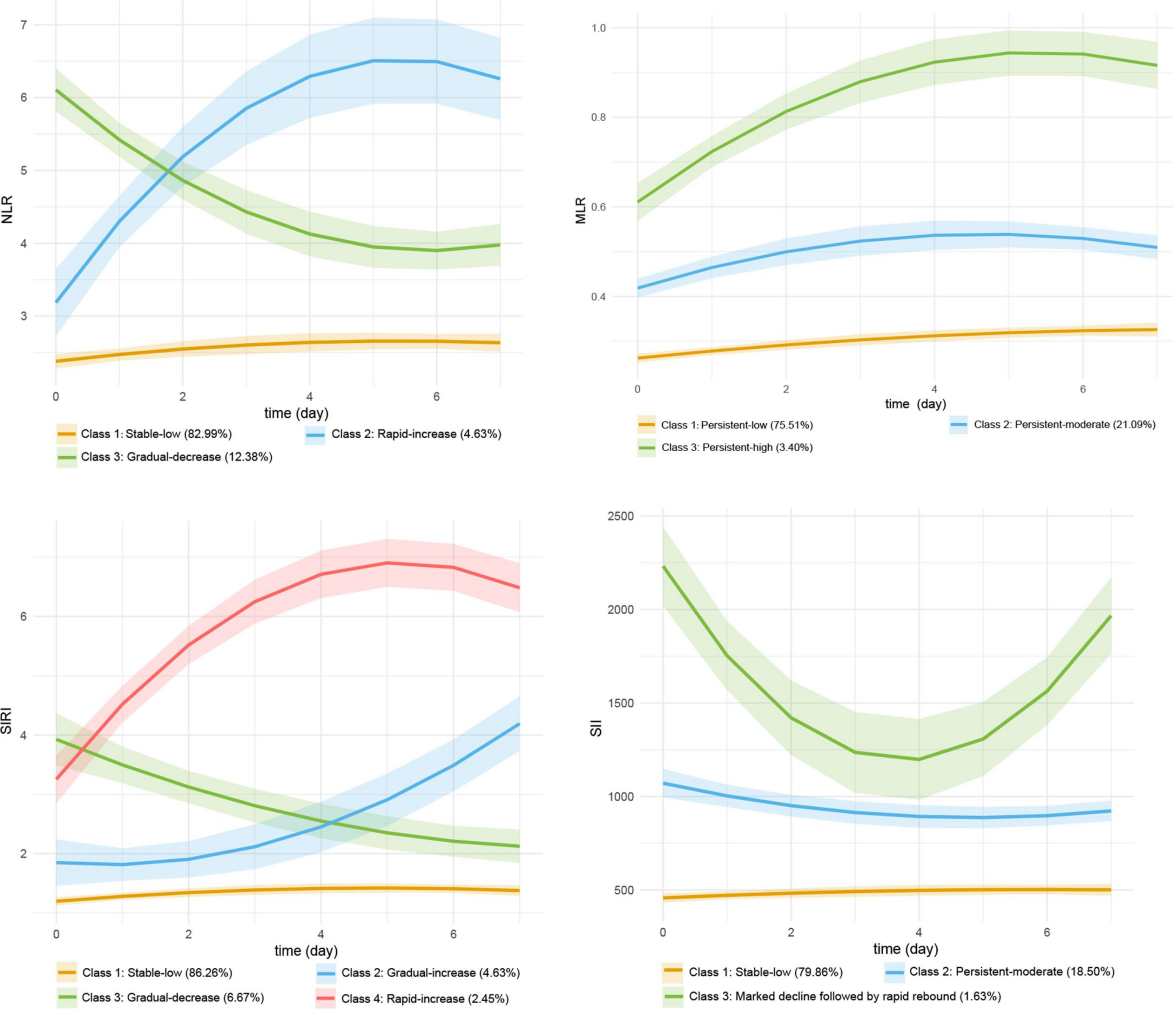
Dynamic trajectories of four inflammatory markers following ischemic stroke. NLR, Neutrophil to Lymphocyte Ratio; MLR, Monocyte to Lymphocyte Ratio; SII, Systemic Immune—Inflammation Index; SIRI, Systemic Inflammatory Response Index.

The temporal trajectories of the four inflammatory markers within the first 7 days are shown in Figure 1. NLR was categorized into three classes: class 1: stable-low (orange trajectory, 82.99%), class 2: rapid-increase (blue trajectory, 4.63%), and class 3: gradual- decrease (green trajectory, 12.38%). MLR also formed three classes: class 1: persistent-low (orange trajectory, 75.51%), class 2: persistent-moderate (blue trajectory, 21.09%), and class 3: persistent-high (green trajectory, 3.40%). SII was divided into three classes: class 1: stable-low (orange trajectory, 79.86%), class 2: persistent-moderate (blue trajectory, 18.50%), and class 3: marked decline followed by rapid rebound (green trajectory, 1.63%). SIRI displayed four distinct classes: class 1: stable-low (orange trajectory, 86.26%), class 2: gradual-increase (blue trajectory, 4.63%), class 3: gradual-decrease (green trajectory, 6.67%), and class 4: rapid-increase (red trajectory, 2.45%). Collectively, these distinct trajectory patterns highlight the marked heterogeneity and temporal variability of systemic inflammation during the 1 week after ischemic stroke.

### 3.3 Baseline and trajectory sub-population characteristics

The baseline characteristics and trajectory subpopulations based on the NLR model are presented in Table 3. The mean age of the 735 participants was 69 years, and 62.6% were male. Overall, 18.6% of cases had an mRS score ≥3 at 3 months. Patients in class 3 were older, had higher baseline NIHSS scores, lower triglycerides, higher blood glucose, and a greater proportion of LAA and CE according to the TOAST classification compared with class 1. Furthermore, patients in class 2 had a higher proportion of mRS ≥ 3 at 3 months than those in class 1.

**Table 3:** Baseline characteristics of three classes based on the NLR model.

Variables	Total (*N* = 735)	Class 1 (*N* = 610)	Class 2 (*N* = 34)	Class 3 (*N* = 91)	*p*
Demographic data					
Age (years), median (IQR)	69 (58, 78)	68 (58, 77)	75 (65, 84)^a^	72 (63, 79)^a^	<0.001
Sex, male, *n* (%)	460 (62.6%)	384 (63.0%)	20 (58.8%)	56 (61.5%)	0.868
BMI, median (IQR)	23.94 (22.04, 26.37)	23.88 (22.05, 26.20)	25.17 (20.92, 27.45)	24.49 (21.63, 26.56)	0.822
Vascular risk factors, *n* (%)					
Hypertension	576 (78.4%)	479 (78.5%)	26 (76.5%)	71 (78.0%)	0.957
Diabetes mellitus	227 (30.9%)	185 (30.3%)	8 (23.5%)	34 (37.4%)	0.254
Ischemic heart disease	109 (14.8%)	85 (13.9%)	7 (20.6%)	17 (18.7%)	0.309
History of stroke/TIA	107 (14.6%)	85 (13.9%)	6 (17.6%)	16 (17.6%)	0.571
Atrial fibrillation	82 (11.2%)	56 (9.2%)	5 (14.7%)	21 (23.1%)^a^	<0.001
Current smoke	186 (25.3%)	157 (25.7%)	8 (23.5%)	21 (23.1%)	0.837
Baseline data					
SBP (mmHg), median (IQR)	155.00 (141.00, 169.00)	155.00 (141.00, 168.00)	153.50 (140.25, 170.25)	158.00 (145.00, 176.00)	0.099
DBP, (mmHg), median (IQR)	87.00 (78.00, 97.00)	86.50 (78.00, 97.00)	83.50 (75.25, 95.00)	88.00 (80.00, 99.00)	0.254
NIHSS, *n* (%)					<0.001
≤3	588 (80.0%)	505 (82.8%)	23 (67.6%)	60 (65.9%)^a^	
4–7	102 (13.9%)	76 (12.5%)	8 (23.5%)	18 (19.8%)^a^	
≥8	45 (6.1%)	29 (4.8%)	3 (8.8%)	13 (14.3%)^a^	
MRS ≥ 3, *n* (%)	137 (18.6%)	100 (16.4%)	14 (41.2%)^a^	23 (25.3%)	<0.001
Intravenous thrombolysis, *n* (%)	110 (15.0%)	91 (14.9%)	8 (23.5%)	11 (12.1%)	0.279
Laboratory data, median (IQR)					
TG (mmol/L)	1.21 (0.87, 1.66)	1.25 (0.92, 1.69)	0.92 (0.61, 1.33)^a^	1.03 (0.74, 1.54)^a^	<0.001
LDL (mmol/L)	2.47 (1.83, 3.12)	2.50 (1.88, 3.17)	2.42 (1.37, 2.93)	2.31 (1.66, 2.98)	0.031
HDL (mmol/L)	1.05 (0.89, 1.24)	1.06 (0.89, 1.24)	1.02 (0.81, 1.18)	1.04 (0.88, 1.30)	0.435
TC (mmol/L)	4.03 (3.36, 4.83)	4.08 (3.43, 4.87)	3.83 (2.96, 4.58)	3.96 (3.06, 4.65)	0.028
Sugar (mmol/L)	6.62 (5.60, 8.58)	6.49 (5.54, 8.48)	6.88 (5.86, 7.99)	7.33 (6.11, 11.39)^a^	0.002
HbA1c (%)	6.00 (5.70, 6.60)	6.00 (5.70, 6.60)	6.15 (5.53, 6.50)	6.10 (5.70, 6.95)	0.352
Toast classification, *n* (%)					<0.001
SAO	391 (53.2%)	345 (56.6%)	16 (47.1%)	30 (33.0%)^a^	
LAA	191 (26.0%)	149 (24.4%)	13 (38.2%)	29 (31.9%)^a^	
CE	62 (8.4%)	41 (6.7%)	3 (8.8%)	18 (19.8%)^a^	
ODE/UE	91 (12.4%)	75 (12.3%)	2 (5.9%)	14 (15.4%)^a^	

^a^Compared with Class 1, *p* < 0.05; All pairwise comparisons were corrected by Bonferroni method.

BMI, Body Mass Index; TIA, Transient Ischemic Attack; SBP, Systolic Blood Pressure; DBP, Diastolic Blood Pressure; NIHSS, National Institutes of Health Stroke Scale; MRS, Modified Rankin Scale; TG, Triglyceride; LDL, Low-Density Lipoprotein; HDL, High-Density Lipoprotein; TC, Total Cholesterol; HbA1c, Glycated Hemoglobin; TOAST classification, Trial of Org 10172 in Acute Stroke Treatment classification; SAO, Small-Artery Occlusion; LAA, Large-Artery Atherosclerosis; CE, Cardioembolism; ODE, Other Determined Etiologies; UE, Undetermined Etiologies; NLR, Neutrophil to Lymphocyte Ratio.

### 3.4 Associations between inflammatory marker trajectories and 3-month outcomes

The associations between inflammatory marker trajectories and 3-month outcomes are presented in Table 4. All independent variables in the logistic regression were assessed using variance inflation factor (VIF) analysis, and all VIF values were <2, indicating no significant multicollinearity. For NLR, class 2 consistently demonstrated an increased risk of poor prognosis across all models (Model 3: OR = 3.492, 95% CI 1.514–10.265), whereas class 3 was significant only in model 1. For MLR, class 3 was associated with poor outcomes in models 1 and 2, but the association was no longer significant in model 3. For SII, class 2 remained significant in models 1 and 2, but not in model 3. For SIRI, both class 2 and class 4 consistently predicted poor prognosis across all models (Model 3: class 2 OR = 3.894, 95% CI 1.395–10.872; class 4 OR = 4.264, 95% CI 1.201–15.136).

**Table 4:** Relationship between inflammatory marker trajectories and 3-month prognosis of cerebral infarction.

Inflammatory markers	Class	Model 1		Model 2		Model 3	
		OR (95%CI)	*p*	OR (95%CI)	*p*	OR (95%CI)	*p*
NLR	Class 1	Reference		Reference		Reference	
	Class 2	3.570 (1.745, 7.304)	<0.05	3.586 (1.730, 7.433)	0.001	3.492 (1.514, 10.265)	<0.05
	Class 3	1.725 (1.026, 2.899)	0.040	1.652 (0.977, 2.793)	0.061	0.721 (0.325, 1.597)	0.420
MLR	Class 1	Reference		Reference		Reference	
	Class 2	1.396 (0.898, 2.169)	0.138	1.437 (0.904, 2.285)	0.126	0.845 (0.447, 1.596)	0.604
	Class 3	3.312 (1.443, 7.599)	0.005	3.490 (1.474, 8.261)	0.004	3.153 (0.956, 10.396)	0.059
SII	Class 1	Reference		Reference		Reference	
	Class 2	2.185 (1.422, 3.359)	<0.05	2.118 (1.370, 3.275)	0.001	1.369 (0.754, 2.486)	0.302
	Class 3	1.748 (0.465, 6.578)	0.409	1.677 (0.441, 6.379)	0.448	1.704 (0.297, 9.769)	0.550
SIRI	Class 1	Reference		Reference		Reference	
	Class 2	4.981 (2.464, 10.070)	<0.05	5.070 (2.476, 10.382)	<0.05	3.894 (1.395, 10.872)	<0.05
	Class 3	0.830 (0.363, 1.898)	0.659	0.789 (0.343, 1.813)	0.577	0.362 (0.106, 1.240)	0.106
	Class 4	3.170 (1.201, 8.364)	0.020	3.424 (1.273, 9.214)	0.015	4.264 (1.201, 15.136)	0.025

Model 1: Unadjusted.

Model 2: Adjustment for age, sex, hypertension, diabetes mellitus.

Model 3: Adjustment for age, sex, hypertension, diabetes mellitus, NIHSS, Systolic Blood Pressure, Intravenous thrombolysis, Low-Density Lipoprotein, HbA1c, Toast classification.

NLR, Neutrophil to Lymphocyte Ratio; MLR, Monocyte to Lymphocyte Ratio; SII, Systemic Immune—Inflammation Index; SIRI, Systemic Inflammatory Response Index; HbA1c, Glycated Hemoglobin; Toast classification, Trial of Org 10172 in Acute Stroke Treatment classification.

## 4 Discussion

In this study, we characterized the temporal dynamics of four inflammatory markers during the acute phase of cerebral infarction and applied LCGM to identify distinct inflammatory trajectories. We further examined the associations between these acute-phase inflammatory trajectories and 3-month functional outcomes. Traditional longitudinal analytical methods, including generalized linear mixed-effects models and generalized estimating equations, are well suited for evaluating overall temporal trends but cannot uncover distinct developmental patterns within heterogeneous populations (18). In contrast, LCGM assumes the presence of unobserved latent subgroups and, through maximum likelihood estimation, simultaneously models unique temporal trajectories for each subgroup while assigning individuals to the most probable class (18, 19). Model selection criteria such as AIC, BIC, entropy, and APP assist in determining the optimal class number (18, 20). In this study, the application of LCGM not only enabled detailed characterization of inflammatory changes during the acute post-stroke period but also allowed investigation of how specific inflammatory trajectories influence functional prognosis, thereby enhancing the clinical relevance of our findings.

Across all inflammatory markers, the persistent or stable low inflammation trajectory group accounted for the largest proportion of patients. During the first 7 days after stroke onset, this trajectory exhibited a slight but gradual increase before reaching a plateau, confirming the activation of the inflammatory response after cerebral infarction (3, 4). Based on NLR, three distinct patterns were identified: stable-low, rapid-increase, and gradual-decrease. Older patients were more likely to exhibit fluctuating inflammatory trajectories (rapid-increase or gradual-decrease), potentially due to impaired immune regulation and increased vulnerability to immune homeostasis disruption (21, 22). Patients with cardioembolic stroke, characterized by abrupt onset and poor collateral circulation (23), often reach peak disease severity immediately, resulting in elevated baseline inflammatory markers (24). Consistent with this, the gradual-decrease group demonstrated the highest baseline inflammatory levels, the highest baseline NIHSS scores, and the greatest proportion of cardioembolic strokes. Notably, triglyceride levels were lower in the fluctuating trajectory groups compared with the stable-low group, possibly reflecting the complex regulatory role of triglycerides in the acute inflammatory response after ischemic stroke (25).

SII trajectories were not associated with clinical outcomes in this study. This may be explained by the fact that platelets are not true inflammatory cells but downstream products of the inflammatory response. Following neuronal necrosis, pro-inflammatory cytokines such as interleukin-6 stimulate hepatocytes to produce thrombopoietic factors, leading megakaryocytes to generate more platelets (26, 27). In addition, routine use of antiplatelet therapy during the acute phase of cerebral infarction can alter platelet function (28), further reducing the ability of platelet-based indices to reflect the intensity of the inflammatory response. In MLR, the trajectories of the three classes—persistent low, persistent moderate, and persistent high—did not intersect. Lymphocytes and monocytes have bidirectional roles in inflammation: B cells and cytotoxic T cells promote pro-inflammatory responses, regulatory T cells suppress excessive inflammation (29); M1-type monocytes drive inflammation, and M2-type monocytes support anti-inflammatory and reparative processes (30). Given this functional complexity, MLR appears to primarily reflect the overall baseline level of immune-inflammatory activation after cerebral infarction, rather than capturing short-term, dynamic fluctuations in inflammatory status.

Among the four inflammatory indices, NLR and SIRI trajectories showed significant associations with 3-month outcomes. Both rapid-increase and gradual-increase patterns were predictive of poor prognosis, whereas the gradual-decrease group, despite higher baseline values, did not show increased risk compared with the stable-low group. These findings suggest that temporal inflammatory patterns, rather than baseline levels alone, may better reflect neurological prognosis. Increased inflammatory marker levels may reflect worsening cerebral edema, hemorrhagic transformation, or infarct expansion, accompanied by a higher risk of neurological deterioration (16, 31). In contrast, declining inflammatory trajectories indicate limited infarct progression and a predominance of anti-inflammatory and reparative processes (11). Interestingly, incorporating monocytes into the NLR framework resulted in the emergence of a gradual-increase trajectory. Compared with neutrophils and lymphocytes, monocytes are activated later in the inflammatory response (32), and this gradual-increase pattern may reflect subacute or chronic inflammation. Within the 7-day observation window, the rapid-increase trajectory showed a declining trend after day 5. However, no downward turning point was observed in the gradual-increase trajectory. Future studies with longer follow-up periods are needed to further characterize the temporal dynamics of monocyte-mediated inflammatory responses.

To our knowledge, this is the first study to evaluate the relationship between dynamic inflammatory trajectories during the acute phase of cerebral infarction and clinical outcomes. By emphasizing temporal inflammatory patterns rather than static baseline measurements, our study addresses limitations of previous work and provides a novel exploratory prognostic framework. Nonetheless, several limitations should be considered. First, infarct volume—an important determinant of DAMP release—was not included in the analysis. Second, the limited number of inflammatory marker measurements prevented adequate modeling of random effects, potentially underestimating within-class heterogeneity. Third, the relatively small sample size within individual trajectory classes limited the ability to perform robust multivariable multinomial logistic regression to identify determinants of inflammatory trajectory membership. Finally, given the exploratory nature of the analysis, as well as the lack of external validation and predictive performance assessment, the clinical application of these findings should be interpreted with caution.

## 5 Conclusion

This study identified distinct early inflammatory trajectories following ischemic stroke. The results further indicate that increasing patterns of NLR and SIRI are strongly associated with an elevated risk of poor functional outcomes at 3 months.

## Data Availability

The raw data supporting the conclusions of this article will be made available by the authors, without undue reservation.
